# Can catheter ablation reduce the incidence of thromboembolic events in patients with atrial fibrillation? Protocol for a systematic review and meta-analysis

**DOI:** 10.1097/MD.0000000000008479

**Published:** 2017-12-01

**Authors:** Menghui Liu, Yuanping Wang, Xiaohong Chen, Xiaohui Li, Xiaodong Zhuang, Lichun Wang

**Affiliations:** a Department of Cardiology, The First Affiliated Hospital, Sun Yet-sen University; b Guangdong Provincial Hospital of Traditional Chinese Medicine affiliated to Guangzhou University of Chinese Medicine; c The Third Affiliated Hospital, Sun Yet-sen University, Guangzhou, China.

**Keywords:** atrial fibrillation, catheter ablation, stroke, systematic review, thromboembolism

## Abstract

Atrial fibrillation (AF), the most common cardiac arrhythmia, is a major risk factor for thromboembolic events, especially ischemic stroke. Catheter ablation is an effective method to maintain sinus rhythm in patients with AF. Although some observational studies have shown a relatively lower stroke rate after catheter ablation, whether catheter ablation can reduce the thromboembolic risk in patients with AF remains unclear. We aim to perform a systematic review to determine whether catheter ablation can prevent thromboembolism in patients with AF.

PubMed, Embase, the Web of Science, and the Cochrane Library will be searched from January 2000 to the present for randomized controlled trials (RCTs) and non-randomized studies on catheter ablation in patients with AF. Other relevant sources, such as the references and conference proceedings, will also be manually retrieved. All studies will be limited to publication in English. The primary outcome will be thromboembolic events, including stroke, transient ischemic attack, and systemic embolic events. Study screening, data collection, and study quality assessment will be independently performed by 2 researchers. Disagreements will be resolved through team discussion or consultation with a third arbitrator. The risk of bias will be appraised using the Cochrane Collaboration tool and the Newcastle–Ottawa scale according to the different study designs, and a meta-analysis will be performed using RevMan V.5.3 software. The results will be presented as risk ratios and 95% confidence intervals for dichotomous data and continuous outcomes.

Catheter ablation is an effective method to cure atrial fibrillation and maintain sinus rhythm. Although it is intuitive that if AF is eliminated, the thromboembolism in the heart would be abolished, and sequently the incidence of thromboembolic events would be decreased, this in fact has not yet been clarified. This systematic review and meta-analysis will be performed with the aim of comprehensively identifying studies that have reported the impact of AF ablation on thromboembolic events in patients with non-valvular AF by comparing an ablation group and non-ablation group. These outcomes will not only produce useful evidence-based data regarding the influence of catheter ablation on thromboembolic events in patients with AF but will also provide some guidance regarding anticoagulation regimens in patients who have undergone catheter ablation.

## 1 Introduction

Atrial fibrillation (AF) is the most common cardiac arrhythmia and an important risk factor for thromboembolic events, especially ischemic stroke.^[1–3]^ The average incidence of ischemic stroke in patients with AF is approximately 5% per year, which is 5-fold higher than that in individuals without AF.^[4,5]^ Recent studies have shown that 20% to 30% of ischemic stroke is related to AF.^[6,7]^ Related research also indicates that thrombosis formation in patients with AF is mainly associated with slow blood flow and stasis of the left atrial appendage secondary to loss of atrial rhythmic mechanical contraction.^[8]^ Based on the mechanism of thrombosis in AF, it is rational to expect that effective rhythm control with either catheter ablation or antiarrhythmic medication might reduce the incidence of thromboembolic events.

Catheter ablation is an effective method to restore and maintain sinus rhythm in patients with non-valvular atrial fibrillation (NVAF).^[9]^ Accumulated data indicate that catheter ablation is even more effective in maintaining sinus rhythm than is antiarrhythmic drug therapy.^[10–12]^ Sinus rhythm can be found after catheter ablation in up to 70% of patients with paroxysmal AF and 50% with persistent AF.^[10,13,14]^ Therefore, as noted above, catheter ablation might reduce thromboembolic events following effective rhythm control. Although some observational studies have shown a relatively lower stroke rate after catheter ablation, whether catheter ablation can reduce the thromboembolic risk remains unclear.^[15–21]^ Consequently, because the true incidence of thromboembolic events after catheter ablation has never been systematically studied, the 2016 European Society of Cardiology Guideline for the management of AF still recommends that “oral anticoagulation after catheter ablation should follow general anticoagulation recommendations, regardless of the presumed rhythm outcome.”^[22]^ Therefore, we designed a systematic review and meta-analysis to compare the incidence of thromboembolic events between patients with NVAF who have and have not undergone catheter ablation. The aim of the review is to elucidate the effects of catheter ablation on thromboembolism.

## 2 Methods and analysis

### 2.1 Inclusion criteria

#### 2.1.1 Types of studies

This review will include randomized controlled trials (RCTs) and non-randomized studies comparing ablation and non-ablation in patients with NVAF with a follow-up duration of at least 6 months. Case reports, review articles, and editorials will be excluded. Trials without detailed results will also be excluded. The language will be restricted to English.

#### 2.1.2 Participants

All included patients will be ≥18 years of age with non-valvular paroxysmal, persistent, or permanent AF. No restrictions on sex or ethnicity will be enforced. Animal studies will be excluded.

#### 2.1.3 Interventions

Patients undergoing radiofrequency catheter ablation will be included as the experimental group. The control group will comprise patients undergoing non-ablation therapy, including rhythm control and rate control with or without antiarrhythmic drugs.

### 2.2 Outcome measures

The primary outcome measure will be the incidence rate of thromboembolic events, including stroke, transient ischemic attack, and systemic embolic events.

### 2.3 Search strategy

#### 2.3.1 Databases

Four English-language databases (PubMed, Embase, the Web of Science, and the Cochrane Library) will be searched without a limitation on publication status. The search strategy will be established according to the Cochrane handbook guidelines. The search terms will be “atrial fibrillation” and “catheter ablation.” This strategy will be utilized to search all eligible studies in English within the above-mentioned databases from January 2000 to the present.

#### 2.3.2 Other resources

In addition to the databases mentioned above, we will manually retrieve papers related to the study topic from other resources, such as conference proceedings and the reference lists of potentially included studies.

### 2.4 Study selection process

**Figure 1:**
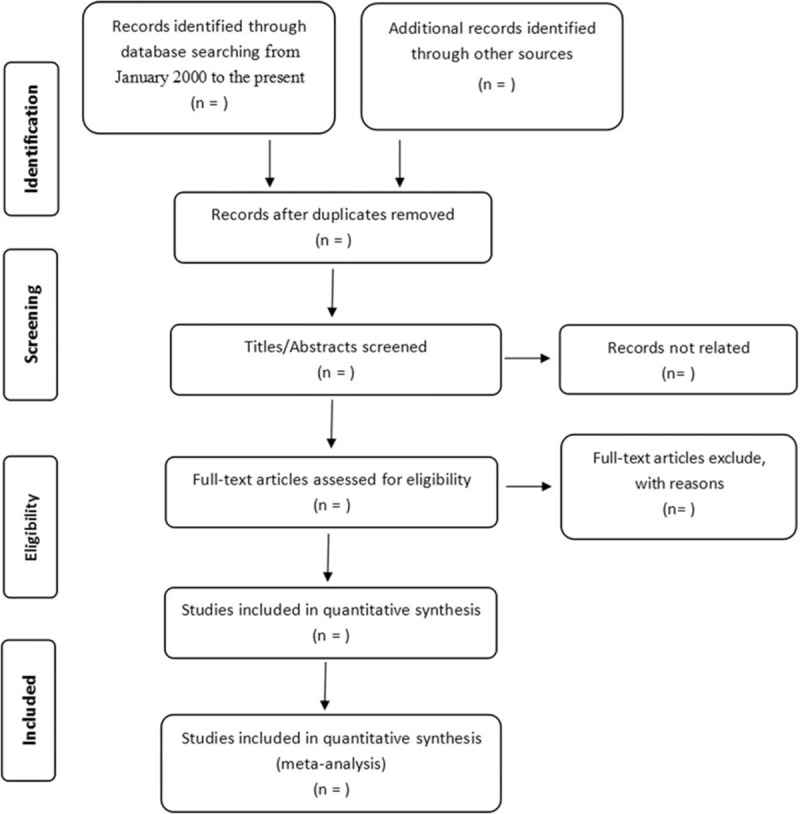
Flow diagram of the study selection process.

All researchers involved in our study have received rigorous training. Two of them will first upload the records retrieved through the electronic or manual search into EndNote software (ver. X7, Thomson Reuters, Philadelphia, Pennsylvania). The titles and abstracts will be independently screened to remove completely unqualified studies, and the full texts of the remaining articles will be reviewed to select eligible studies. If possible, we will contact the authors to obtain the full texts and clarify the data for the studies with insufficient information. Detailed reasons for exclusion of any studies will be provided. During this process, any conflicts will be resolved by team discussion; if necessary, a third investigator will make the final decision. The details of the selection process are shown in the flow diagram in Figure 1.

### 2.5 Data extraction and management

Two authors will independently extract data from the eligible studies by double entry and checking using EpiData Software, version 3.1 (EpiData Association, Odense, Denmark). We will abstract the basic characteristics and outcome data of each study, including information regarding the authors, year of publication, research design, sample size, follow-up, type of AF, thromboembolic events, age, sex, medical history, CHA_2_DS_2_ score, CHA_2_DS_2_-VASc score, and other parameters. If the information reported in the studies is insufficient, we will attempt to contact the authors. During this process, any disagreements will be resolved by group discussion between 2 researchers or arbitration by a third researcher.

### 2.6 Quality assessment of risk of bias

The risk of bias or methodological quality of all included studies will be independently assessed by 2 researchers. We will appraise the methodological quality of non-randomized studies using the Newcastle–Ottawa scale.^[23]^ The Cochrane Collaboration's tool will be used for RCTs.^[24]^ When disagreements occur during this appraisal, we will resolve the conflicts through team discussion or consultation with a third arbitrator.

### 2.7 Dealing with missing data

In cases of missing data, we will attempt to contact the authors of the original study to request the missing or incomplete data by telephone or e-mail. If this fails, we will perform a further analysis based on the available data to determine the potential impact of the missing or incomplete data on the study quality.

### 2.8 Data synthesis and analysis

RevMan version 5.3 software (The Cochrane Collaboration, Oxford, England) will be used for data analysis, and the results will be shown as risk ratios with 95% confidence intervals and *P* values. Heterogeneity between groups will be investigated by the χ^2^ statistic and *I*^2^ statistic. According to the Cochrane Handbook for Systematic Reviews of Interventions, studies are considered to have no statistical heterogeneity when *P *≥ .1 and *I*^2^ ≤ 50%; in such cases, we will use a fixed-effect model to perform the analysis.^[25]^ However, significant differences among trials are considered to be present when *P* < .1 and *I*^2^ > 50%; in such cases, we will use a random-effects model. Considering that some factors, such as the CHA_2_DS_2_ score, type of AF, and so on, are likely to lead to outcome bias, we will perform a subgroup analysis for these factors. When significant heterogeneity is identified between the included studies, a sensitivity analysis will be performed through removal of individual studies. In addition, funnel plots will be used to assess the reporting bias if more than 10 studies are included in the meta-analysis.

### 2.9 Grading the quality of evidence

We will classify the quality of the evidence as high, moderate, low, or very low in accordance with the Grading of Recommendations Assessment, Development and Evaluation (GRADE) to assess the quality of all outcomes.^[26]^

### 2.10 Ethics and dissemination

Ethical approval is not required for this systematic review because the data are not individual and the review involves no privacy issues. We will disseminate the protocol of this systematic review in a peer-reviewed journal or through conference presentations. The completed review will later be disseminated electronically and in print.

### 2.11 Trials status

We have already begun to search the 4 English-language databases (PubMed, Embase, the Web of Science, and the Cochrane Library) since August 20, 2017, and we intend to complete this systematic review and meta-analysis in January 2018. Registration number: PROSPERO CRD 42017056636.

### 2.12 Data sharing statement

The technical appendix, statistical code, and dataset will be available from the corresponding author at Dryad repository, who will provide a permanent, citable, and open access home for the dataset.

### 2.13 Protocol amendments

If it is necessary to amend the protocol, the authors will provide a detailed explanation of the date, change, and rationale.

## 3 Discussion

**Figure 2:**
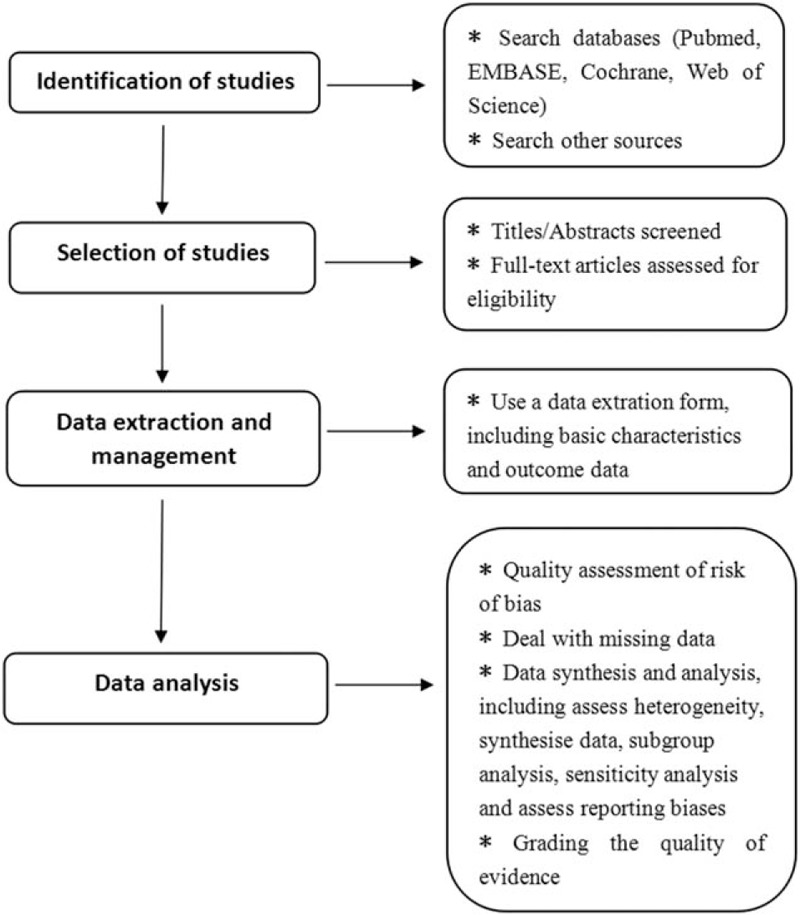
Flow diagram of the systematic review and meta-analysis.

The aim of AF ablation is to reduce the burden of AF and maintain sinus rhythm. Based on the mechanism of thrombosis in patients with AF, catheter ablation should simultaneously reduce thromboembolic events in these patients. However, whether catheter ablation can reduce the risk of thromboembolic events in patients with AF remains unclear. Therefore, a high-quality systematic review and meta-analysis is necessary, and the process is shown in the flow diagram in Figure 2. This systematic review and meta-analysis will be performed with the aim of comprehensively identifying studies that have reported the impact of AF ablation on thromboembolic events in patients with NVAF by comparing an ablation group and non-ablation group. These outcomes will provide some guidance regarding anticoagulation regimens in patients who have undergone catheter ablation. We will also perform subgroup analyses to identify factors that may influence the effects of catheter ablation on thromboembolic events in patients with AF. We anticipate that the results of this review will not only produce useful evidence-based data regarding the influence of catheter ablation on thromboembolic events in patients with AF but will also identify some variances that may affect this relationship. Such information will be useful for future studies and clinical anticoagulation therapy after AF ablation.

This systematic review might have some limitations. First, the incidence of thromboembolic events after catheter ablation has not been systematically studied to date, and most clinical trials are observational studies, which would affect the level of evidence of the outcomes. Second, because of the absence of a standard method of catheter ablation for AF, especially persistent AF, the methods used may differ among studies, even among the individual patients within the same study. It is not possible to consider the diversity of the catheter ablation method in this analysis. This might have some bias on the results. Furthermore, although we will search 4 major English-language databases that will include most of the relevant studies, we will still probably miss some original research articles, which could limit the broadness of the search and affect the outcomes.

### 3.1 Strengths and limitations of this study

This systematic review and meta-analysis will focus on the exploration of the impact of ablation of atrial fibrillation on thromboembolic events.

The review will provide useful evidence-based guidance for the treatment of atrial fibrillation.

The review will include both randomized controlled trials and non-randomized studies, and the language will be restricted to English.

This systematic review protocol was designed in accordance with the preferred reporting items for systematic review and meta-analysis protocols (PRISMA-P) statement, and the review will follow the Grading of Recommendations Assessment, Development and Evaluation (GRADE) approach for assessing the quality of the evidence.

## Acknowledgments

We thank Angela Morben, DVM, ELS, from Liwen Bianji, Edanz Group China (www.liwenbianji.cn/ac), for editing the English text of a draft of this manuscript.
